# Comparing the values of intact parathormone and 1– 84 PTH to predict hyperparathyroidism in hemodialysis patients

**DOI:** 10.15171/jnp.2017.40

**Published:** 2017-04-22

**Authors:** Aria Jenabi, Mosadegh Jabbari, Hossein Ziaie

**Affiliations:** Nephrology ward, Rasoul Akram Medical Center, Iran University of Medical Sciences Tehran Iran

**Keywords:** Intact parathormone, iPTH, 1-84 PTH, ESRD, Hemodialysis, Bio-active PTH, Chronic kidney disease

## Abstract

**Background:**

Secondary hyperparathyroidism (SHPT) is a common complication of chronic kidney disease (CKD) leading high mortality and even long-term morbidity. SHPT is manifested by elevation of parathyroid hormone (PTH) and accurate determining the level of serum PTH is very essential for early diagnosis of SHPT secondary to CKD. It is very important to match the values obtained for intact parathormone (iPTH) and 1– 84 PTH with the minimized measurement bias.

**Objectives:**

The present study aimed to first determine the agreement value between the iPTH and 1– 84 PTH measures in patients with hyperparathyroidism secondary to endstage renal disease under chronic hemodialysis. Then, we attempted to determine the best cutoff values for these two measurements for detecting SHPT in such patients.

**Patients and Methods:**

This cross-sectional study was conducted on hemodialysis patients. The value of study biomarkers including iPTH and 1– 84 PTH was assessed.

**Results:**

A strong positive association was revealed between the two indicators of iPTH and 1-84 PTH (r = 0.800, *P* < 0.001). The linear association between these two parameters is independent to baseline characteristics including gender, age, body mass index, and medical history. Among all biochemical elements, the value of 1-84 PTH was only associated with serum calcium level negatively (r = -0.267, *P* = 0.027) and alkaline phosphatase positively (r = 0.359, *P* = 0.003). Considering iPTH as the reference and according to the area under the ROC curve (AUC), 1-84 PTH had high value to predict hyperparathyroidism (AUC = 0.926, *P* < 0.001). The best cutoff point for 1-84 PTH to discriminate hyperparathyroidism from normal condition was 60 yielding a sensitivity of 92.3% and a specificity of 79.1%. Among other baseline laboratory parameters, only alkaline phosphatase had an acceptable value for diagnosing hyperparathyroidism (AUC = 0.731, *P* = 0.001).

**Conclusions:**

The measurement of both iPTH and 1-84 PTH is valuable for predicting hyperparathyroidism secondary to CKD, but according to lower cost and comparableeffectiveness of iPTH measurement, this assay may be comparable to 1-84 PTH to predict this consequence.

**Implication for health policy/practice/research/medical education:**

The measurement of both iPTH and 1-84 PTH is valuable for predicting hyperparathyroidism secondary to CKD, but according to lower cost and comparable-effectiveness of iPTH measurement, this assay may be comparable to 1-84 PTH to predict this consequence.

## 1. Background

Chronic kidney disease (CKD) is a common cause of death and significant disability worldwide so that only in the United States, about 6 million people suffer from kidney dysfunction and of them, nearly 400000 need to dialysis (1-4). Secondary hyperparathyroidism (SHPT) is a common complication of CKD leading high mortality and even long-term morbidity. SHPT is manifested by a defined triad including elevation of parathyroid hormone (PTH), vitamin D deficiency, and impairment of mineral metabolism (5). SHPT is the result of progressive CKD leading cardiovascular disorders and renal osteodystrophy leading high mortality, high treatment costs as well as prolonged hospitalization. In other word, SHPT can be caused by any abnormal change in maintaining mineral homeostasis by the kidneys. In patients with progressing CKD, developing disease stage leads to depression of vitamin D level along with increase of PTH level (6,7). Thus, significant elevation in serum PTH concentration as a major marker for SHPT is predicted in patients with CKD that a PTH level higher than >300 pg/mL can be detected in about one-fourth of CKD patients (8,9) that can be accompanied with cardiac and skeletal diseases in long-term period. Therefore, accurate determining the level of serum PTH is very essential for early diagnosis of SHPT secondary to CKD.

After producing early inactive form of PTH as prepro-PTH in parathyroid glands, it is cleaved to pro-PTH and then to an active form of hormone named 1–84 PTH or intact PTH (iPTH). Within stimulation of parathyroid glands activating in response to calcium and phosphorus changes, this active form of PTH metabolite to its fragments (10,11). It has been shown that the accuracy of PTH assays for measurement of 1– 84 PTH is closely associated with the degree of 1– 84 PTH to cross reactivity with these fragments (12). First generations of these assays had cross reactivity with various types of fragments. Second-generation PTH immunoassays could better detect 1–84 PTH, but may react only to long C-terminal PTH fragments (13). In third-generation PTH immunoassays, higher specificity for 1–84 PTH assessment was achieved. The main advantage of the latter assays was to determine iPTH and 1–84 PTH with high accuracy in both healthy individuals and CAD patients (14-16). However, it is very important to match the values obtained for iPTH and 1–84 PTH with the minimized measurement bias.

## 2. Objectives

The present study aimed to first determine the agreement value between the iPTH and 1–84 PTH measures in patients with SHPT secondary to CKD. Then, we attempted to determine the best cutoff values for these two measurements for detecting SHPT in such patients.

## 3. Patients and Methods

This cross-sectional study (as a test value assessment assay) was conducted on patients with CKD who underwent hemodialysis in Rasoul-e-Akram general hospital in Tehran between 2014 and 2015. All patients were aged higher than 20 years with the history of dialysis longer than 3 months. On admission, the informed consent was taken from all participants. A venous blood sample was obtained before dialysis and transferred to a single laboratory to measure the value of study biomarkers including iPTH, 1–84 PTH, and elements of Ca, P, creatinine, albumin, hemoglobin, alkaline phosphatase, and 25-hydroxy vitamin D levels. A checklist was also applied to collect baseline characteristics and medical history of patients by interviewing. The level of 1–84 PTH was determined by the chemiluminescence assay and the level of iPTH by ELISA method with the normal reference values of 5-58 pg/mL and 10.4-66.5 pg/mL, respectively. The serum level of 25-hydroxy vitamin D was determined too. Additionally, to determine the cases with effective hemodialysis, Kt/V parameter was calculated. To diagnose SHPT, the cutoff value of 400 pg/mL was considered for iPTH. Accordingly, patients with SHPT were assessed by Tc-99m MIBI SPECT too. Among those patients, only two were finally diagnosed as parathyroid adenoma, one at left lower lobe and another at right lower lobe of the glands.

### 3.1. Ethical issues

1) The research followed the tenets of the Declaration of Helsinki; 2) informed consent was obtained; and 3) This study was approved by the Ethics Committee of Iran University of Medical Sciences.

### 3.2. Statistical analysis

For statistical analysis, results were presented as mean ± standard deviation (SD) for quantitative variables and were summarized by frequency (percentage) for categorical variables. Continuous variables were compared using *t* test or Mann-Whitney U test whenever the data did not appear to have normal distribution or when the assumption of equal variances was violated across the study groups. Categorical variables were, on the other hand, compared using chi-square test. The association between the quantitative variables, the Pearson’s or Spearman’s tests was applied. The area under the ROC analysis was measured to determine the value of both iPTH and 1–84 PTH to predict SHPT. The agreement between iPTH and 1–84 PTH values was assessed using the Kappa agreement test. *P* values of ≤ 0.05 were considered statistically significant. For the statistical analysis, the statistical software SPSS version 23.0 for windows (IBM, Armonk, New York) was used.

## 4. Results

In total, 70 patients (47 men and 23 women, mean age 62.32 ±13.42 years, ranged 14 to 83 years) with CKD under hemodialysis were assessed. The baseline characteristics are summarized in Table 1. The mean duration of dialysis was 44 months as 3 to 4 hours in each session with 3 sessions per week. Regarding underlying risk factors, 51.4% were diabetic, 64.3% were hypertensive, and 25.7% were smoker. The most common etiology for renal failure was shown to be simultaneous history of diabetes and hypertension (27.1%). Considering the serum levels of biomarkers showed hypocalcaemia in 17.1%, hyperphosphatemia in 65.3%, vitamin D deficiency in 80.0%, and vitamin D insufficiency in 18.5%. Based on the value of KT/V ratio, effective dialysis was found in 70.0% of patients undergoing dialysis.

**Table 1:** Baseline characteristics of study participants

**Characteristics**	
Gender	
Men	47 (67.1)
Women	23 (32.9)
Age (year)	62.32 ± 13.42
Body mass index, kg/m^2^	24.41 ± 4.42
Duration of dialysis, month	44 ± 4
Time of dialysis in each session	
3 h	8 (11.4)
3.5 h	25 (35.7)
4 h	37 (52.9)
History of diabetes	36 (51.4)
History of hypertension	45 (64.3)
History of smoking	18 (25.7)
Etiology of renal failure	
Concurrent diabetes and hypertension	19 (27.1)
Diabetes alone	18 (25.7)
Hypertension alone	16 (22.9)
Glomerulonephritis	8 (11.4)
Polycystic kidney disease	4 (5.7)
Renal stones	2 (2.9)
Vasculitis	1 (1.4)
Hypocalcaemia	12 (17.1)
Hyperphosphatemia	45 (65.3)
Vitamin D deficiency	56 (80.0)
Vitamin D insufficiency	13 (18.5)
Ineffective dialysis	21 (30.0)

**Figure 1:**
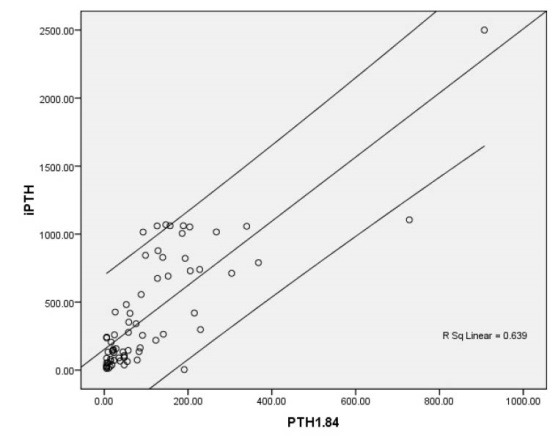
Association between iPTH and 1-84 PTH.

The mean level of iPTH was 409.16 ± 446.27 pg/mL and the mean level of 1-84 PTH was also 111.32 ± 151.64 pg/mL. Overall, 37.1% of patients suffered hyperparathyroidism. A strong positive association was revealed between the two indicators of iPTH and 1-84 PTH (r = 0.800, *P* < 0.001) (Figure 1). As shown in Table 2, the linear association between these two parameters is independent to baseline characteristics including gender, age, body mass index, and medical history. Among all biochemical elements, the value of 1-84 PTH was only associated with serum calcium level negatively (r = -0.267, *P* = 0.027) and alkaline phosphatase positively (r = 0.359, *P* = 0.003), but not with vitamin D or phosphorus levels.

**Table 2:** Association between iPTH and 1-84 PTH according to different subgroups

	*P value*
Gender	
Men	0.830
Women	0.650
Age group	
≤ 70 years	0.806
> 70 years	0.681
Obesity	
Present	0.915
Absent	0.793
Diabetes	
Present	0.893
Absent	0.657
Hypertension	
Present	0.675
Absent	0.894
Smoking	
Present	0.826
Absent	0.797

**Figure 2:**
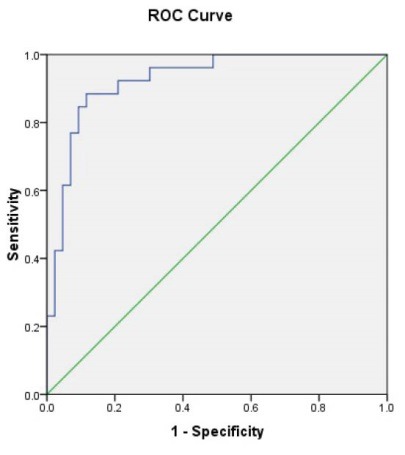
The area under the ROC curve for assessment of the value of 1-84 PTH for predicting hyperparathyroidism.

**Figure 3:**
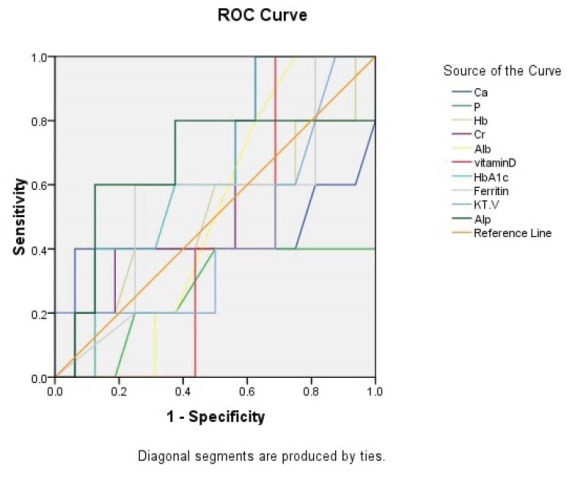
The area under the ROC curve for assessment of the value of different laboratory parameters for predicting hyperparathyroidism.

Considering iPTH as the reference and according to the area under the ROC curve (Figure 2), 1-84 PTH had high value to predict hyperparathyroidism (AUC = 0.926, *P* < 0.001). The best cutoff point for 1-84 PTH to discriminate hyperparathyroidism from normal condition was 60 yielding a sensitivity of 92.3% and a specificity of 79.1% (Figure 1). Also, we found that the difference between the two parameters (iPTH and 1-84 PTH) had high value for diagnosis of hyperparathyroidism (AUC = 0.995, *P* < 0.001) that in a cutoff value of 200 pg/mL could predict hyperparathyroidism with sensitivity and specificity of 100% and 86%, respectively; however, iPTH to 1-84PTH ration could not predict hyperparathyroidism (AUC = 0.370, *P* = 0.073). Also, two ratios of (iPTH – 1-84 PTH/ iPTH) and (iPTH – 1-84 PTH/1-84 PTH) had no enough power to predict hyperparathyroidism (AUC of 0.630 for both) (Table 3). Among other baseline laboratory parameters, only alkaline phosphatase had an acceptable value for diagnosing hyperparathyroidism (AUC = 0.731, *P* = 0.001; Figure 3).

**Table 3:** The area under the ROC curve to predict hyperparathyroidism using different parameters

**Parameter**	
iPTH, Pg/mL	1
1-84 PTH, Pg/mL	0.926
1-84 PTH/iPTH	0.370
1-84 PTH – iPTH	0.995
(1-84 PTH – iPTH)/iPTH	0.630
(1-84 PTH – iPTH)/1-84PTH	0.630
Alp, IU/L	0.731
Ca, mg/dL	0.425
P, mg/dL	0.269
Alb, g/dL	0.500
Hb, g/dL	0.500
Cr, mg/dL	0.612
Vitamin D, ng/mL	0.413
HBA1C, %	0.644
Ferritin, ng/mL	0.550
Kt/V, %	0.438

## 5. Discussion

Early prediction of appearing hyperparathyroidism in patients with chronic renal insufficiency is very critical due to its related irreversible consequences such as cardiovascular disorders or deterioration of renal failure. The occurrence of SHPT can be predicted by assessing serum PTH level and its fragments within activation. However the assessment of these fragments can be achieved more accurate assessment of hyperparathyroidism in such patients. In this regard, considering both efficacy and cost-effectiveness of these tests is also necessary especially in populations of the developing countries. Hence, the present study aimed to assess the value of two assessment assays of iPTH and 1-84 PTH for predicting hyperparathyroidism secondary to chronic renal failure to recommend the assay with higher cost-effectiveness. First, we showed a strong association between iPTH and 1-84 PTH indicating high efficiency of both measurements two predict hyperparathyroidism. However due to considerably low cost of measuring iPTH as compared to 1-84PTH, the former assay can be already recommended to predict hyperparathyroidism and its related metabolites alterations. Moreover, according to the association of serum creatinine level with iPTH but not with 1-84PTH, the assessment of iPTH can better detect non-function fragment such as (7-84)PTH in kidney failure especially ESRD condition that is not detected by new third generation bioactive PTH. Another point was that the diagnostic value of both assays was not affected by baseline patients’ characteristics such as gender, age, body mass index or medical history indicating high value of these two indicators for assessing the probability for hyperparathyroidism in background of renal failure regardless of baseline confounding factors.

Almost all previous literatures obtained similar findings with respect to association between iPTH and 1-84 PTH. In a study by Melamed et al (17), two indices were strongly correlated with together. In this regard, the best cutoff value of 1-84 PTH for predicting patients’ death was estimated to be 160 pg/mL. In our study, the cutoff value of 60 pg/mL was found to predict occurrence of hyperparathyroidism in such patients. In a study by Tan et al (18), Elecsys PTH (1-84) was strongly associated with iPTH as well as with serum level of alkaline phosphatase as similarly obtained in our study. In another study by O’Flaherty et al (19), however, the level of iPTH was significantly higher than 1-84 PTH. In a study by Hecking et al (20), not only two assays had strong association, but also iPTH was strongly associated with serum phosphorus and creatinine. Finally, it can be concluded that the measurement of both iPTH and 1-84 PTH is valuable for predicting hyperparathyroidism secondary to chronic renal failure, but because of lower cost and good effectiveness of iPTH measurement, this assay can be considered comparable to 1-84 PTH measurement to predict this consequence.

## Limitations of the study

This study was conducted on a limited proportion of patients and we suggest larger studies on this feature of hemodialysis patients.

## Acknowledgements

We would like to express our appreciation to the full co-operation of the Rasoul Akram dialysis center staff in collecting the data and also head of Jam-e-Jam lab center -Korosh Asadi - for performing lab data.

## Authors’ contribution

AJ contributed to design of research and edited the manuscript. AJ and MJ conducted the experiments and research and HZ collected and analyzed the data and prepared primary draft.

## Conflicts of interest

There were no points of conflicts.

## Funding/Support

This project was extracted from residential thesis of Hossein Ziaie affiliated to Iran University of Medical Sciences.
